# A simulation-based optical safety framework for dermatologic laser wavelength selection across Fitzpatrick skin phototypes

**DOI:** 10.1007/s10103-026-04922-4

**Published:** 2026-08-08

**Authors:** Nur Ecer, Ömer Karakoyun, Kadir Kaya

**Affiliations:** 1 Gazi Yaşargil Training and Research Hospital, Diyarbakır, Türkiye; 2 Istinye University, Istanbul, Turkey

**Keywords:** Laser therapy, Skin phototype, Monte Carlo simulation, Dermatology, Wavelength selection, Optical safety

## Abstract

Laser wavelength selection in dermatology remains challenging across diverse skin phototypes, where inappropriate choice may increase the risk of epidermal injury. This study develops a simulation-based optical safety framework to support wavelength selection by quantifying phototype-dependent epidermal energy deposition and dermal-to-epidermal energy balance. A Monte Carlo light-transport model was used to simulate photon propagation in a three-layer skin model across Fitzpatrick skin types I–VI. Four commonly used dermatologic wavelengths (532, 755, 808 and 1064 nm) were evaluated. Epidermal energy deposition was quantified using the Epidermal Thermal Risk Index (ETRI), and dermal-to-epidermal energy balance was quantified using the Benefit–Risk Ratio (BRR). ETRI categories were defined as heuristic interpretive ranges. A composite score combining safety and energy-balance components was computed and tested across multiple weighting schemes via sensitivity analysis. Shorter wavelengths produced markedly higher epidermal energy deposition, particularly in higher phototypes. At 532 nm, ETRI rose monotonically with melanin content, indicating elevated epidermal optical burden. In contrast, 1064 nm maintained the lowest ETRI values across all phototypes. Across five tested weighting schemes, including extreme configurations, 1064 nm consistently demonstrated the highest composite score in every phototype (Spearman ρ = 1.00 against the equal-weight baseline), supporting robustness of the framework. This study presents a simulation-based optical safety framework that translates wavelength-dependent energy deposition into an interpretable structure for laser wavelength selection. Within the assumptions of the model, longer wavelengths — and 1064 nm in particular — demonstrate the most favorable epidermal safety profile and dermal-to-epidermal energy balance across higher phototypes. The framework is intended as relative optical safety guidance and must be integrated with treatment indication, target chromophore, fluence, pulse duration, epidermal cooling, device-specific settings and operator experience in clinical practice.

**Supplementary Information:**

The online version contains supplementary material available at 10.1007/s10103-026-04922-4.

## Introduction

Laser and light-based technologies have become fundamental tools in dermatology, with widespread applications in hair removal, vascular and pigmented lesion treatment, and skin rejuvenation. These procedures rely on the principle of selective photothermolysis, in which specific chromophores absorb optical energy and produce controlled thermal injury of targeted structures while sparing surrounding tissue [1]. Despite their broad utility, treatment outcomes and safety profiles vary significantly with patient-specific factors, particularly skin pigmentation.

Skin phototype, commonly classified using the Fitzpatrick scale, plays a critical role in determining laser–tissue interactions [2]. Melanin, the primary epidermal chromophore, exhibits strong wavelength-dependent absorption, which increases the risk of non-specific epidermal heating in higher phototypes (IV–VI). Clinically, this has been associated with adverse effects such as burns, post-inflammatory hyperpigmentation, hypopigmentation, and scarring in darker skin types [3–5]. Hypopigmentation is of particular concern, as melanin is an efficient chromophore and disproportionate epidermal absorption may lead to permanent pigmentary loss; pulsed dye laser treatment in Fitzpatrick types IV–VI, for example, has been reviewed as carrying an increased risk of pigmentary adverse events including both hyper- and hypopigmentation [6]. Wavelength selection is therefore a key determinant of both safety and efficacy in dermatologic laser applications.

Longer wavelengths, particularly in the near-infrared range (e.g., 1064 nm), are generally preferred for treating patients with increased melanin content due to their lower absorption by epidermal melanin and deeper tissue penetration [7, 8]. However, current clinical decision-making remains largely experience-based and lacks quantitative, standardized tools to support parameter selection across different skin phototypes and treatment scenarios. This gap highlights the need for objective frameworks that translate underlying optical principles into clinically interpretable guidance.

Computational modeling, particularly Monte Carlo simulation of photon transport, has been widely used to investigate light–tissue interactions and predict energy distribution within biological tissues [9, 10]. These models enable systematic evaluation of wavelength-dependent absorption and scattering effects under controlled conditions, offering a powerful approach to study phototype-specific differences that are difficult to isolate experimentally. Existing studies, however, often focus on isolated wavelengths or limited skin types and rarely provide clinically interpretable metrics for decision support.

In this study, we present a simulation-based optical safety framework for laser wavelength selection across Fitzpatrick skin phototypes. Using a Monte Carlo model, we quantify epidermal energy deposition across commonly used dermatologic wavelengths (532, 755, 808 and 1064 nm). We introduce a normalized metric, the Epidermal Thermal Risk Index (ETRI), and combine it with a complementary dermal-to-epidermal energy-balance metric, the Benefit–Risk Ratio (BRR), within a multi-objective composite score. The framework is positioned as relative optical safety guidance to complement, not replace, clinical judgement and indication-specific parameter optimization.

## Materials and methods

### Skin optical model

A three-layer planar tissue model was constructed to represent the epidermis, dermis and subcutaneous tissue. This simplified geometry was selected to isolate the primary variable of interest — melanin-dependent epidermal absorption — while maintaining computational efficiency. The model assumes homogeneous optical properties within each layer and does not explicitly account for microstructural heterogeneity such as discrete melanocyte distribution, vascular networks or adnexal structures.

Layer thicknesses and optical parameters were assigned based on previously published literature [11–13]. Epidermal thickness was set between 60 and 100 μm; dermal and subcutaneous layers were modeled at 2.0 mm and 5.0 mm, respectively. Optical properties included the absorption coefficient (µₐ), reduced scattering coefficient (µₛ′), anisotropy factor (g) and refractive index (n), all defined as wavelength-dependent where applicable.

### Melanin absorption model

Epidermal absorption was modeled as a combination of melanin and baseline tissue absorption:

$$\mu_a=f\_mel\;\cdot\;\mu\_mel\;+\;\left(1-f\_mel\right)\;\cdot\;\mu\_baseline$$

Melanin absorption followed a wavelength-dependent power-law relationship [14]:

$$\mu\_mel\;=\;6.6\;\times\;10^{11}\;\cdot\;\lambda^\wedge\left(-3.33\right)$$

Melanin volume fractions corresponding to Fitzpatrick skin types were assigned based on experimental measurements reported in the literature [15], ranging from 1.3% (Type I) to 35% (Type VI).

### Monte Carlo simulation

Photon transport was simulated using a Monte Carlo method based on the MCML framework [16]. Simulations were implemented in Python (version 3.9) with Numba acceleration to improve computational efficiency.

A flat-top circular beam with a diameter of 6 mm was used to approximate clinical laser delivery conditions. Photons were launched perpendicular to the tissue surface and propagated through the layered medium using probabilistic sampling of scattering and absorption events.

The simulation domain measured 10 × 10 × 7.1 mm with a voxel resolution of 50 μm. Scattering was modeled using the Henyey–Greenstein phase function. Boundary conditions included Fresnel reflection at the air–tissue interface and internal reflections between tissue layers.

Each simulation consisted of 2 × 10⁶ photon packets. For each condition, five independent runs were performed and results were averaged to ensure numerical stability.

### Evaluated wavelengths and phototypes

Four clinically relevant laser wavelengths were evaluated: 532, 755, 808 and 1064 nm. These wavelengths cover the principal melanin- and pigment-targeting range used in dermatologic practice. Pulsed dye laser wavelengths (e.g., 595 nm), which are primarily used for vascular indications and target hemoglobin rather than melanin, were not included in the present analysis. Their inclusion would require a chromophore-specific vascular model and is identified as a direction for future work (Section 4). Simulations were conducted across all six Fitzpatrick skin phototypes (I–VI), enabling systematic assessment of wavelength-dependent optical energy distribution as a function of melanin content.

### Epidermal thermal risk index (ETRI)

To quantify epidermal optical burden, the Epidermal Thermal Risk Index (ETRI) was defined as:

$$ETRI\;=\;\left(E\_epidermis/E\_total\right)\;\times\;100$$

where E_epidermis is the absorbed energy within the epidermal layer and E_total is the total absorbed energy across all tissue layers.

ETRI provides a normalized metric enabling direct comparison across wavelengths and phototypes independent of absolute fluence. Three heuristic interpretive categories were defined to facilitate visualization and stratification:

- Lower epidermal burden: ETRI < 30%.
- Intermediate epidermal burden: 30% ≤ ETRI ≤ 50%.
- Higher epidermal burden: ETRI > 50%.

These thresholds were selected as pragmatic interpretive categories to facilitate visualization and comparative risk stratification across the simulated dataset, rather than as biologically validated thresholds for clinical injury. The 50% boundary was chosen to denote conditions in which epidermal deposition becomes the dominant component of total simulated energy deposition. ETRI should not be interpreted as a direct probability of clinical complications; it is a relative optical-burden indicator under controlled simulation assumptions, and validation against thermal modeling and clinical outcomes is required before any direct clinical translation.

### Multi-objective optimization framework

#### Benefit–risk ratio (BRR)

To complement ETRI, the Benefit–Risk Ratio (BRR) was defined as the ratio of dermal to epidermal absorbed energy:

$$BRR\;=\;E\_dermis/E\_epidermis$$

**Note on terminology.** Although the metric is named the *Benefit–Risk Ratio* for consistency with our earlier work, the term *benefit* here refers strictly to dermal-to-epidermal optical energy balance and **does not imply direct clinical efficacy**. Throughout this manuscript, BRR is treated as an optical energy-balance metric only, and its interpretation in the context of indication-specific target chromophores is discussed in Section 4.1.

Higher BRR values indicate a more favorable distribution of optical energy, characterized by greater dermal energy deposition relative to epidermal absorption. Lower BRR values indicate predominant epidermal absorption.

It is important to emphasize that BRR is an optical energy-balance metric, not a direct measure of clinical efficacy. Therapeutic efficacy depends on absorption by the indication-specific target chromophore (e.g., follicular melanin in hair removal, dermal pigment in pigmentary lesions, hemoglobin in vascular lesions, exogenous tattoo pigment, or other lesion-specific structures). A wavelength with a favorable BRR may therefore not be the most effective option for every indication. BRR should be interpreted as a phototype-dependent indicator of dermal-to-epidermal energy distribution that complements indication-specific clinical reasoning.

#### Composite scoring and sensitivity analysis

To enable comparative evaluation across conditions, ETRI and BRR were normalized to the [0, 1] range across the full dataset. A composite score was defined as:

$$Score\;=\;w_1\;\cdot\;\left(1-ETRI\_norm\right)\;+\;w_2\;\cdot\;BRR\_norm$$

where w₁ and w₂ represent weighting coefficients for the safety and energy-balance components, respectively. Equal weighting (w₁ = w₂ = 0.5) was used as the baseline configuration.

To assess robustness of the composite score to weighting assumptions, a sensitivity analysis was performed using four alternative weighting schemes in addition to the baseline: safety-prioritized (0.70 / 0.30), penetration-prioritized (0.30 / 0.70), extreme safety (0.90 / 0.10) and extreme penetration (0.10 / 0.90). For each scheme, the optimal wavelength per phototype and the full wavelength ranking were recomputed. Spearman rank correlations were used to compare alternative-scheme rankings against the baseline. This analysis was added in response to peer review and is reported in Section 3.4 and Supplementary Table S1.

### Decision support structure

ETRI categories, BRR values and composite scores were combined into a structured comparative table presenting relative wavelength performance across phototypes. The structure is intended as relative optical safety guidance to support — not replace — clinical decision-making, which must integrate treatment indication, target chromophore, fluence, pulse duration, cooling, device-specific delivery profile, operator experience and individual patient factors.

### Sensitivity analysis of optical parameters

In addition to weighting-scheme sensitivity, a one-way sensitivity analysis was performed to evaluate the influence of key optical parameters on model outputs. Melanin volume fraction (± 20%), epidermal thickness (± 25%) and reduced scattering coefficient (± 30%) were varied independently. Changes in ETRI and BRR were quantified to assess parameter sensitivity.

### Statistical analysis

Results are reported as mean ± standard deviation across five independent simulation runs. The coefficient of variation (CV) was calculated to assess numerical stability. Because simulations are deterministic under fixed conditions, variability reflects computational precision rather than biological variability.

## Results

### Wavelength-dependent epidermal energy deposition

**Fig. 1:**
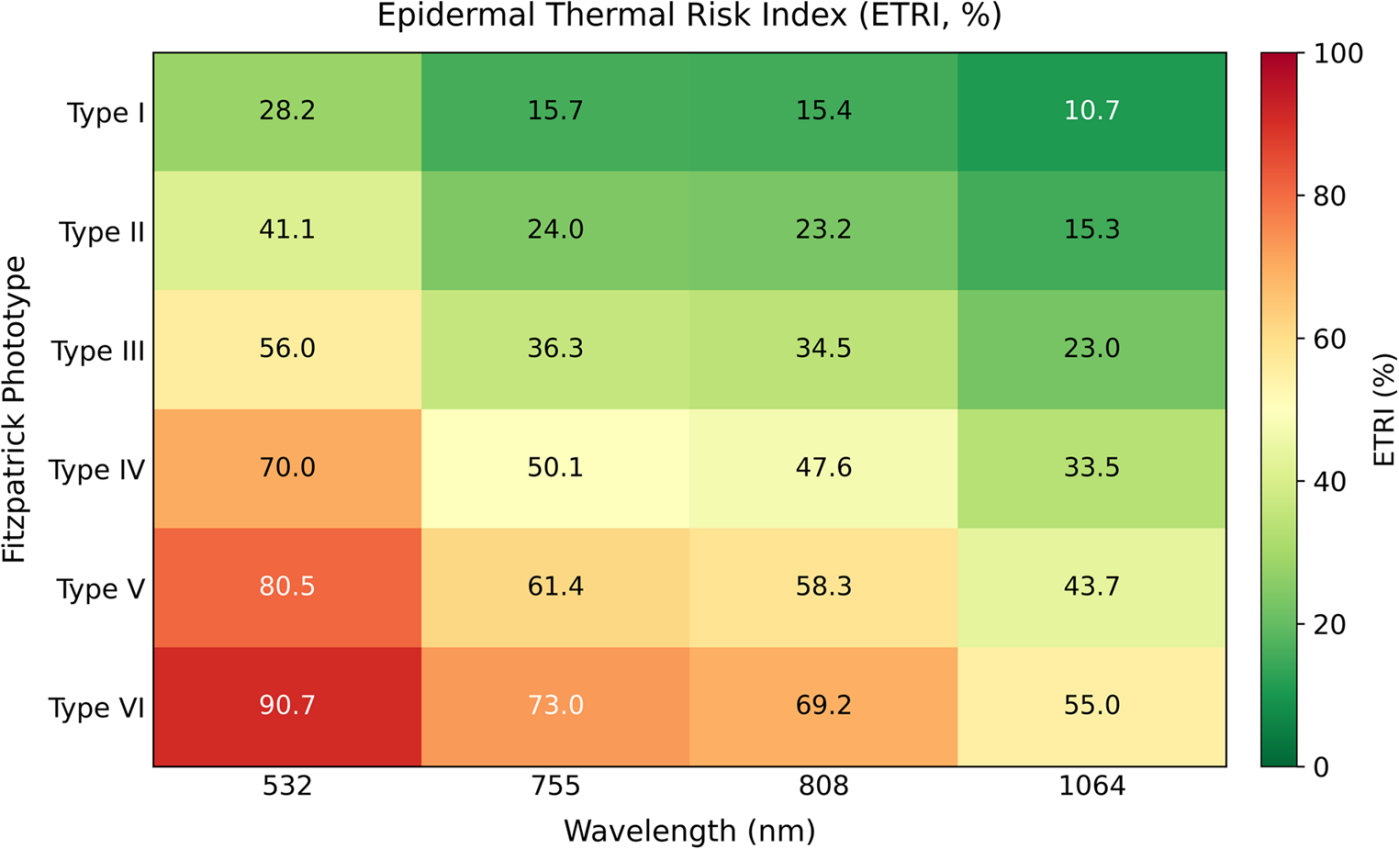
Heat-map visualization of ETRI (%) across wavelengths and Fitzpatrick phototypes. Color scale ranges from green (lower epidermal burden) to red (higher epidermal burden)

Epidermal energy deposition, quantified by the Epidermal Thermal Risk Index (ETRI), demonstrated strong dependence on both wavelength and skin phototype (Table 1; Fig. 1). ETRI values ranged from 10.7% (Type I at 1064 nm) to 90.7% (Type VI at 532 nm), an approximately eight-fold variation across the evaluated conditions.

**Table 1:** Epidermal thermal risk index (ETRI, %) across wavelengths and phototypes

Phototype	532 nm	755 nm	808 nm	1064 nm	Burden Trend
I	28.2 ± 0.13	15.7 ± 0.06	15.4 ± 0.09	10.7 ± 0.04	Lower
II	41.1 ± 0.10	24.0 ± 0.16	23.2 ± 0.08	15.3 ± 0.05	Lower–Intermediate
III	56.0 ± 0.22	36.3 ± 0.18	34.5 ± 0.07	23.0 ± 0.09	Intermediate
IV	70.0 ± 0.15	50.1 ± 0.20	47.6 ± 0.09	33.5 ± 0.19	Intermediate–Higher
V	80.5 ± 0.08	61.4 ± 0.12	58.3 ± 0.08	43.7 ± 0.14	Higher
VI	90.7 ± 0.12	73.0 ± 0.20	69.2 ± 0.12	55.0 ± 0.25	Higher

*Values are mean ± SD (n = 5 independent runs). Color shading reflects heuristic interpretive categories: green (< 30%)*,* yellow (30–50%)*,* red (> 50%). Categories are simulation-derived interpretive ranges*,* not validated clinical thresholds.*

At 532 nm, ETRI increased progressively with melanin content, from 28.2% in Type I to 90.7% in Type VI, indicating marked confinement of optical energy within the epidermis in darker skin types. Phototypes IV–VI exceeded the higher-burden interpretive threshold (> 50%) at this wavelength, with Type VI exhibiting the highest epidermal energy burden across all conditions.

In contrast, 1064 nm maintained the lowest ETRI values across all phototypes, ranging from 10.7% (Type I) to 55.0% (Type VI). Notably, even in Type VI the ETRI at 1064 nm (55.0%) was comparable to values observed at shorter wavelengths in intermediate phototypes (e.g., Type III at 532 nm: 56.0%), indicating that wavelength selection can substantially modify epidermal optical burden across the pigmentation spectrum.

Intermediate wavelengths (755 nm and 808 nm) showed similar ETRI profiles, with moderate increases across phototypes. At 808 nm, ETRI remained below 50% for Types I–IV, transitioning to higher-burden values only in Types V (58.3%) and VI (69.2%).

### Dermal energy deposition and benefit–risk ratio

**Fig. 2:**
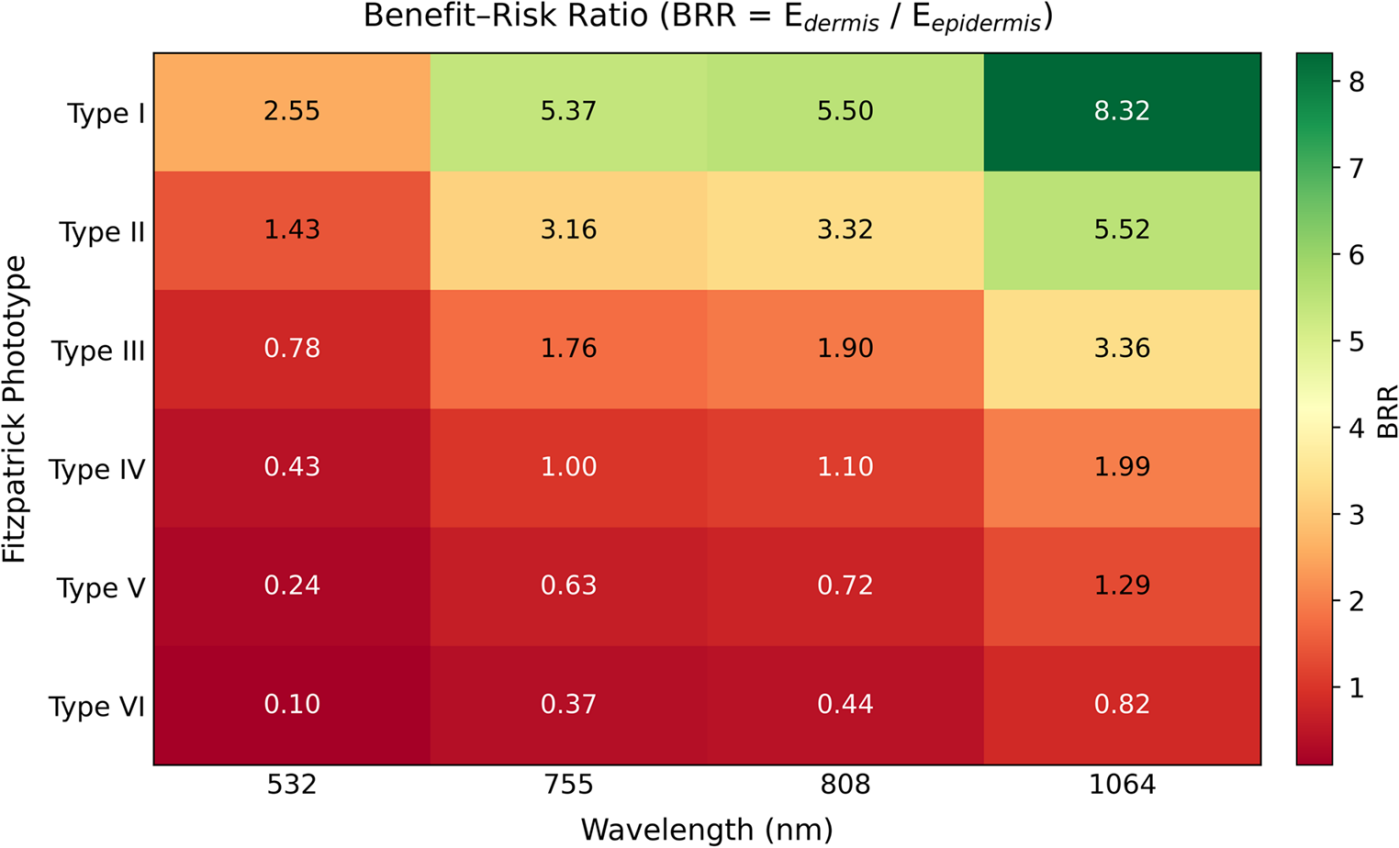
Heat-map visualization of benefit–risk ratio (BRR) across wavelengths and phototypes. Higher values (green) indicate more favorable dermal-to-epidermal energy balance

The Benefit–Risk Ratio (BRR), defined as the ratio of dermal to epidermal energy deposition, provided a complementary metric for evaluating wavelength performance (Table 2; Fig. 2). Higher BRR values indicate more favorable energy distribution, with greater dermal penetration relative to epidermal absorption.

**Table 2:** Benefit–risk ratio (BRR = E_dermis / E_epidermis) across wavelengths and phototypes

Phototype	532 nm	755 nm	808 nm	1064 nm
I	2.55 ± 0.02	5.37 ± 0.03	5.50 ± 0.04	8.32 ± 0.03
II	1.43 ± 0.01	3.16 ± 0.03	3.32 ± 0.01	5.52 ± 0.02
III	0.78 ± 0.01	1.76 ± 0.01	1.90 ± 0.01	3.36 ± 0.02
IV	0.43 ± 0.00	1.00 ± 0.01	1.10 ± 0.00	1.99 ± 0.02
V	0.24 ± 0.00	0.63 ± 0.00	0.72 ± 0.00	1.29 ± 0.01
VI	0.10 ± 0.00	0.37 ± 0.00	0.44 ± 0.00	0.82 ± 0.01

*Values are mean ± SD (n = 5 independent runs). BRR > 1 indicates greater dermal than epidermal energy deposition.*

BRR demonstrated substantial variation across conditions, ranging from 0.10 (Type VI at 532 nm) to 8.32 (Type I at 1064 nm). At 1064 nm, BRR remained above 0.82 across all phototypes, indicating near-equivalent or greater dermal energy deposition relative to epidermal absorption even in highly pigmented skin. In contrast, at 532 nm, BRR values fell below 1.0 for Types III–VI, indicating predominant epidermal confinement of optical energy.

The inverse relationship between BRR and melanin content was most pronounced at shorter wavelengths. At 532 nm, BRR decreased by a factor of approximately 25 from Type I (2.55) to Type VI (0.10). At 1064 nm, the reduction was more modest, with BRR decreasing by a factor of approximately 10 across the same phototype range (8.32 to 0.82). As noted in Section 2.6.1, BRR reflects dermal-to-epidermal optical energy balance and is not a direct measure of clinical efficacy, which depends on indication-specific target chromophore absorption.

### Composite score and wavelength comparison

**Fig. 3:**
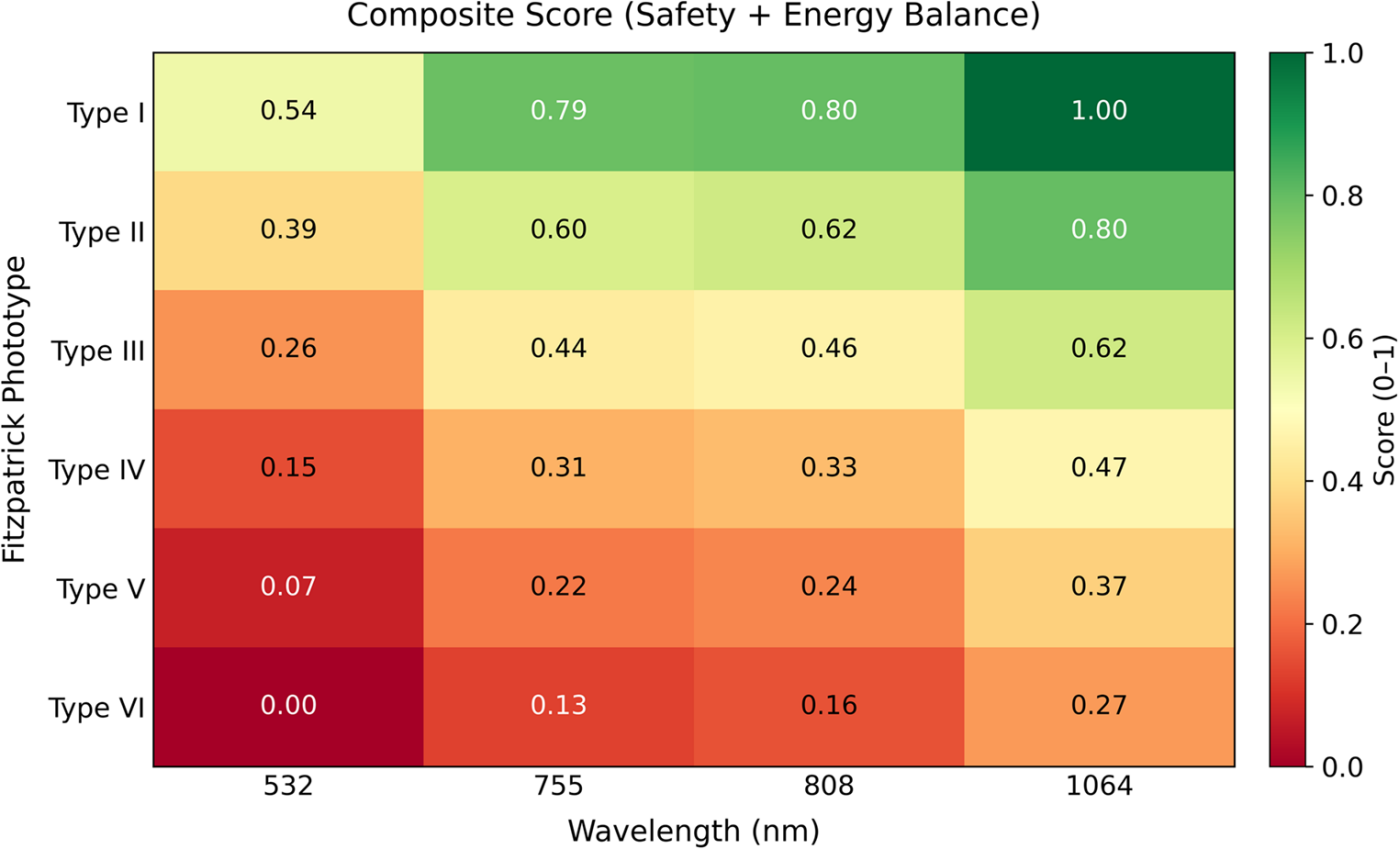
Composite score heat-map integrating safety (ETRI) and energy-balance (BRR) components. Higher scores (green) indicate a more favorable overall optical profile

Integration of ETRI and BRR into a composite score enabled unified evaluation of wavelength performance considering both safety and energy-balance components (Table 3; Fig. 3). The composite score ranged from 0 (least favorable) to 1 (most favorable), with equal weighting assigned to normalized safety (1 − ETRI_norm) and energy-balance (BRR_norm) components in the baseline configuration.

**Table 3:** Composite score (baseline weighting w₁ = w₂ = 0.5) and most favorable wavelength by phototype

Phototype	532 nm	755 nm	808 nm	1064 nm	Most Favorable
I	0.54	0.79	0.80	1.00	1064 nm
II	0.39	0.60	0.62	0.80	1064 nm
III	0.26	0.44	0.46	0.62	1064 nm
IV	0.15	0.31	0.33	0.47	1064 nm
V	0.07	0.22	0.24	0.37	1064 nm
VI	0.00	0.13	0.16	0.27	1064 nm

*Composite Score *=* 0.5 × (1 − ETRI_norm) + 0.5 × BRR_norm. Higher scores indicate a more favorable balance of epidermal safety and dermal-to-epidermal energy distribution. Green shading marks the highest-scoring wavelength per phototype.*

Across all phototypes, 1064 nm achieved the highest composite score in the baseline weighting, ranging from 1.00 (Type I) to 0.27 (Type VI). This finding supports — within the assumptions of the model — the use of 1064 nm as the wavelength with the most favorable epidermal safety profile and dermal-to-epidermal energy balance across the full Fitzpatrick range.

For lower phototypes (I–III), multiple wavelengths produced acceptable composite scores, with values above 0.40 for all wavelengths except 532 nm in Type III. For higher phototypes (IV–VI), the gap between 1064 nm and shorter wavelengths widened substantially. In Type VI, the composite score for 1064 nm (0.27) was approximately twice that of 808 nm (0.16) and more than twenty-fold higher than 532 nm (0.00).

### Sensitivity of composite score to weighting scheme

To address the choice of equal weighting in the baseline configuration, a sensitivity analysis was performed across five weighting schemes (Section 2.6.2; Supplementary Table S1; Supplementary Figure S1). In addition to the baseline (0.50 / 0.50), the analysis included safety-prioritized (0.70 / 0.30), penetration-prioritized (0.30 / 0.70), extreme safety (0.90 / 0.10) and extreme penetration (0.10 / 0.90) configurations.

Across all five schemes, 1064 nm achieved the highest composite score in every Fitzpatrick phototype. The full wavelength ranking (1064 > 808 > 755 > 532) was preserved under every scheme, including the extreme configurations. Spearman rank correlation between alternative-scheme rankings and the baseline was 1.00 in all phototypes for all alternative schemes. The directional conclusions of the framework are therefore robust to the choice of weighting and not driven by the equal-weight assumption.

In practice, weighting may be adapted to reflect indication-specific priorities (for example, prioritizing safety in higher phototypes treated for benign cosmetic indications, or prioritizing dermal penetration in deeper-target indications). The robustness of the wavelength ranking across weighting configurations supports use of the framework as a general-purpose optical safety reference, while preserving flexibility for indication-specific re-weighting.

### Comparative optical safety structure

Based on the integrated analysis of ETRI, BRR and composite scores, a comparative optical safety structure was constructed (Table 4). Three relative-suitability categories were defined as heuristic interpretive ranges based on the composite score: more favorable (composite score ≥ 0.50), use with caution (0.25–0.49) and less favorable (< 0.25). These categories are simulation-derived interpretive ranges and represent relative wavelength comparisons within the model, not absolute clinical recommendations or contraindications.

**Table 4:** Relative optical safety structure for wavelength comparison across phototypes

Phototype	532 nm	755 nm	808 nm	1064 nm
I	More favorable	More favorable	More favorable	More favorable
II	Use with caution	More favorable	More favorable	More favorable
III	Use with caution	Use with caution	Use with caution	More favorable
IV	Less favorable	Use with caution	Use with caution	Use with caution
V	Less favorable	Less favorable	Less favorable	Use with caution
VI	Less favorable	Less favorable	Less favorable	Use with caution

*Categories are heuristic interpretive ranges based on the composite score under the baseline weighting (more favorable: ≥0.50; use with caution: 0.25–0.49; less favorable: <0.25). Categories represent relative optical-safety positioning within the model and are not absolute clinical recommendations. Wavelength selection in clinical practice must additionally consider indication*,* target chromophore*,* fluence*,* pulse duration*,* epidermal cooling*,* device-specific delivery*,* operator experience and patient-level factors.*

### Summary of key findings

The principal findings can be summarized as follows: (1) ETRI demonstrated an approximately eight-fold variation across evaluated conditions, with highest values at 532 nm in Type VI (90.7%) and lowest at 1064 nm in Type I (10.7%); (2) BRR values indicated that 1064 nm produced the most favorable dermal-to-epidermal energy balance across all phototypes; (3) the composite score, evaluated under five weighting schemes including extreme configurations, consistently identified 1064 nm as the highest-scoring wavelength in every phototype, with rank ordering invariant to weighting; and (4) the comparative optical safety structure provides a relative reference for wavelength comparison, supporting reduced reliance on shorter wavelengths in higher phototypes when other parameters are not otherwise constrained by indication-specific requirements.

## Discussion

This study presents a simulation-based optical safety framework for laser wavelength selection across Fitzpatrick skin phototypes. By integrating epidermal optical burden (ETRI) with dermal-to-epidermal energy balance (BRR), and assessing robustness through weighting-scheme sensitivity analysis, we provide a multi-objective quantitative reference structure that complements clinical experience-based decision-making. The principal finding is that, within the assumptions of the model, 1064 nm consistently demonstrates the most favorable epidermal safety profile and dermal-to-epidermal energy distribution across all evaluated phototypes.

The clinical implications of these findings are substantial, particularly for higher phototypes (IV–VI), where the model supports preferential consideration of longer wavelengths, especially 1064 nm [17–19]. At 1064 nm, even Type VI skin maintained a composite score of 0.27, compared to near-zero scores at 532 nm. This quantitative differentiation provides an objective optical-safety reference that complements clinical experience and may be particularly useful for practitioners with limited experience treating diverse skin types. The framework reinforces, with quantitative optical modeling, a long-standing clinical teaching point: longer wavelength devices, particularly the 1064 nm Nd: YAG, exhibit favorable epidermal safety across the full Fitzpatrick range, while shorter-wavelength devices warrant greater caution in higher phototypes [17–19].

Our findings are consistent with established principles of selective photothermolysis and clinical observations regarding laser safety in skin of color [1, 2]. The marked increase in ETRI at shorter wavelengths in higher phototypes aligns with the wavelength-dependent absorption characteristics of melanin and the clinically observed higher incidence of pigmentary adverse effects, including hypopigmentation, post-inflammatory hyperpigmentation and burns, in darker skin types [3–6, 17–19]. Previous Monte Carlo studies have characterized light transport in skin tissue [9, 10] but typically focused on single wavelengths or limited phototype ranges. The present study extends prior work by systematically evaluating four clinically relevant wavelengths across all six Fitzpatrick phototypes and, importantly, by translating simulation outputs into an interpretable comparative structure. The combination of ETRI, BRR and weighting-scheme sensitivity testing represents a methodological refinement, enabling simultaneous consideration of safety and energy distribution rather than risk assessment alone.

### Interpreting BRR in indication-specific practice

The Benefit–Risk Ratio introduced here provides a perspective complementary to risk-focused metrics, but is intentionally limited to optical energy balance. BRR quantifies dermal-to-epidermal energy distribution, which favors wavelengths that deliver more energy to the dermis while sparing the epidermis. It does not, however, quantify absorption by the indication-specific target chromophore. In indications where the therapeutic target is hemoglobin (e.g., port-wine stains, telangiectasias), pulsed dye laser at 595 nm remains the established first-line treatment due to selective hemoglobin absorption, despite its shorter wavelength [20]. In such cases, a wavelength with a less favorable BRR may nevertheless be the preferred clinical choice. BRR should therefore be interpreted in conjunction with — not in place of — indication-specific chromophore considerations.

The dramatic variation in BRR across conditions — from 0.10 (Type VI at 532 nm) to 8.32 (Type I at 1064 nm) — does, however, highlight the substantial impact of wavelength choice on optical energy distribution. In Type VI skin, choosing 1064 nm over 532 nm corresponds to roughly an eight-fold improvement in dermal-to-epidermal energy balance, an effect that is meaningful for indications targeting deeper or melanin-related structures.

### Robustness of the composite score

A key methodological consideration is the choice of weighting in the composite score. Rather than asserting a single weighting as definitive, we tested robustness across five configurations, including extreme cases. The wavelength ranking (1064 > 808 > 755 > 532) was preserved across all weighting schemes for every phototype, with Spearman correlations of 1.00 between alternative-scheme rankings and the baseline. This robustness indicates that the framework’s directional conclusions are not contingent on a specific weighting and that practitioners can adapt weighting to indication-specific priorities without altering the relative ordering of wavelengths under the model.

### Clinical implementation

The comparative optical safety structure (Table 4) is intended as a relative reference, not an absolute prescription. In clinical practice, it can be used in two primary ways. First, as an at-a-glance comparator when selecting between wavelengths for indications in which multiple devices are available — for example, when choosing between 755 nm alexandrite and 1064 nm Nd: YAG for laser hair reduction in a Type IV–V patient, the structure highlights the lower epidermal burden and more favorable energy balance of 1064 nm under the model. Second, as a teaching and risk-communication aid, providing a quantitative basis for the long-standing clinical caution against shorter wavelengths in higher phototypes. In all cases, the structure must be combined with indication-specific requirements (e.g., target chromophore), device-specific delivery parameters (fluence, pulse duration, spot size, repetition rate, cooling), patient-specific factors (medical history, prior pigmentary response, treatment goals) and operator experience. Adjustable parameters — particularly epidermal cooling, longer pulse durations and conservative fluences — can substantially modify the actual clinical safety profile of shorter wavelengths in higher phototypes [21], a factor that the present optical-only framework does not capture.

### Limitations

Several limitations warrant emphasis. First, the model evaluates wavelength under standardized optical assumptions and does not explicitly model fluence, pulse duration, epidermal cooling, spot size, repetition rate or device-specific delivery profiles. In clinical practice, these parameters substantially influence both safety and efficacy. Epidermal cooling in particular can reduce surface thermal injury and enables shorter wavelengths such as 755 nm and 808 nm to be used safely in selected higher phototypes [21]. The ETRI values reported here therefore should be interpreted as relative optical-burden indicators under controlled simulation assumptions and not as direct probabilities of clinical complications; the present framework may overestimate epidermal risk relative to optimally-cooled clinical delivery.

Second, the model employs a simplified three-layer tissue geometry that does not account for microstructural heterogeneity, including discrete melanocyte distribution, vascular networks or adnexal structures. Third, melanin volume fractions were assigned based on population averages and may not reflect within-phototype individual variation. Fourth, the simulation does not incorporate temporal dynamics of heat diffusion or thermal relaxation, which are critical determinants of actual tissue injury [22]. Fifth, the framework focuses on melanin-targeted applications and does not include hemoglobin-targeted vascular indications; pulsed dye laser at 595 nm, in particular, is widely used clinically for vascular lesions and could not be meaningfully evaluated within the present melanin-centered optical model. Sixth, the ETRI interpretive thresholds (30%, 50%) and the composite-score categories (0.25, 0.50) are heuristic interpretive boundaries adopted for visualization and stratification within this dataset; they are not validated against clinical injury outcomes. Seventh, the simulation outputs have not been validated against prospective clinical data.

Together, these limitations imply that the framework should be interpreted as a structured optical-safety reference rather than a clinical decision instrument. Translation to clinical practice requires integration with thermal modeling, indication-specific chromophore modeling and prospective clinical validation.

### Future directions and validation strategy

Several directions emerge from this work. (1) Integration of thermal modeling, including heat-diffusion and damage-integral computations (e.g., Arrhenius-based thermal damage models [22]), would enable prediction of temperature distributions and tissue-injury thresholds, linking optical deposition to clinical injury risk. (2) Inclusion of epidermal cooling and pulse-duration modeling would extend the framework to capture parameters that materially affect clinical safety. (3) Expansion of the wavelength range to include hemoglobin-targeted wavelengths (e.g., 595 nm) with chromophore-specific vascular modeling would broaden applicability to vascular indications. (4) Indication-specific extensions — incorporating absorption by follicular melanin, dermal pigment, hemoglobin or exogenous chromophores such as tattoo pigment — would enable target-aware composite scoring. (5) Prospective clinical validation studies, correlating model-predicted optical-safety categories with measured pigmentary outcomes (post-inflammatory hyperpigmentation, hypopigmentation, burns) across phototype-stratified cohorts, would establish the predictive value of the framework. (6) Multi-objective weighting could be extended to incorporate additional clinical objectives, such as treatment depth or chromophore selectivity. Such extensions would enhance the clinical utility of simulation-based decision support in dermatologic laser applications.

## Conclusion

This study presents a simulation-based optical safety framework for laser wavelength selection across Fitzpatrick skin phototypes. By integrating epidermal optical burden assessment (ETRI) with dermal-to-epidermal energy balance (BRR), and demonstrating robustness through weighting-scheme sensitivity analysis, we provide a quantitative reference for wavelength comparison that captures both safety and energy-distribution considerations within the assumptions of an optical model.

The findings quantitatively support the established clinical principle that longer wavelengths generally reduce relative epidermal optical burden in higher Fitzpatrick phototypes. Among the evaluated wavelengths, 1064 nm showed the most favorable model-derived epidermal safety profile and dermal-to-epidermal energy balance, and this ordering was robust across all tested weighting schemes. The Benefit–Risk Ratio provides a complementary optical energy-balance metric, while the comparative optical safety structure offers a relative reference for wavelength comparison rather than absolute clinical guidance.

These results should therefore be interpreted as simulation-based optical safety guidance within the assumptions of the model. Real-world laser selection must integrate treatment indication, target chromophore, fluence, pulse duration, epidermal cooling, device-specific settings, operator experience and patient-level factors. Future studies incorporating thermal modeling, indication-specific chromophore absorption and prospective clinical validation are needed to translate this framework into routine practice.

## Supplementary Information

Below is the link to the electronic supplementary material.

Supplementary Material 1 (XLSX 6.32 KB)

Supplementary Material 2 (PNG 234 KB)

## Data Availability

The simulation code and data are available at: https://github.com/omar-k2025/A-Simulation-Based-Decision-Support-Framework-for-Laser-Parameter-Selection-Across-Skin-Phototypes. DOI: 10.5281/zenodo.19427750.

## Code availability

Monte Carlo simulation code (Python/Jupyter) available under MIT License at the repository above.
